# Feasibility and Safety of Food Containing *Acanthopanax* *senticosus* for Treating Patients with Cancer-Related Fatigue

**DOI:** 10.1089/pmr.2024.0041

**Published:** 2024-08-23

**Authors:** Yutaka Kawano, Nanae Watanabe, Masahiko Nishiyama, Tousei Ohmura, Hiroyoshi Mihara, Kaoru Ono, Maki Tanaka, Yasushi Sato, Tetsu Tomonari, Hidekatsu Takeda, Tetsuji Takayama

**Affiliations:** ^1^ Department of Community Medicine and Medical Science, Tokushima University Graduate School of Biomedical Sciences, Tokushima, Japan.; ^2^ Department of Gastroenterology and Oncology, Tokushima University Graduate School of Biomedical Sciences, Tokushima, Japan.; ^3^ Higashi Sapporo Hospital, Sapporo, Japan.; ^4^ Department of Molecular Pharmacology and Oncology, Gunma University Graduate School of Medicine, Maebashi, Japan.; ^5^ Department of Clinical Laboratory Science, School of Medical Technology, Health Sciences University of Hokkaido, Sapporo, Japan.; ^6^ Department of Physical Therapy, Sapporo Medical University School of Medicine, Sapporo, Japan.

**Keywords:** *Acanthopanax senticosus* Harms, cancer-related fatigue, quality of life, adaptogen

## Abstract

**Background:**

Cancer-related fatigue (CRF) is a major obstacle to quality of life. *Acanthopanax senticosus* Harms (ASH) is available as a botanical adaptogen food worldwide.

**Objective:**

This study aimed to assess the feasibility and safety of ASH in patients with CRF.

**Methods:**

Fifteen patients with CRF consumed ASH drink for 28 days. The primary endpoint was the completion rate of the study, and the secondary endpoints were changes in brief fatigue inventory (BFI), oxidative stress markers, and adverse events.

**Results:**

Seven patients successfully completed the study. Four patients who had BFI <5.5 at enrollment revealed a decrease in BFI. The biological antioxidant potential/diacron-reactive oxygen metabolites ratio, potential antioxidant capacity, was increased but not significant (*p* = 0.063). No adverse events attributable to ASH were observed.

**Conclusions:**

Approximately 50% patients were successful in consuming ASH for 28 days. Patients with mild CRF showed improvement by using ASH. However, further investigations are needed to validate these findings.

## Background

Approximately half of the people in Japan are diagnosed with cancer during their lifetime.^1^ When diagnosed with cancer at an early stage and treated curatively, it has little impact on patients’ prognosis. However, advanced cancer that is difficult to treat by radical resection or refractory to chemotherapy and radiation would pose a threat to patient survival. Patients with advanced cancer also suffer from a sense of fatigue and malaise, called cancer-related fatigue (CRF).^2^ A meta-analysis reported that 60.9% of patients with advanced cancer were experiencing CRF,^3^ which is associated with both patients’ quality of life and daily activities.^4^ CRF is caused by several factors, including tumor growth, pain, anemia, poor nutrition, deconditioning, depression, and cancer therapy.^5^ Inflammatory cytokines (IL-6, TGF-β)^6^ and an increased level of oxidative stress^7^ are also known to cause CRF. Even though glucocorticoids,^8^ antidepressants, and Chinese herbal medicines such as Bojungikki-Tang (Hochu-ekki-to)^9^ have been used, no treatment has been established for CRF.^10^

Ezoukogi (*Acanthopanax senticosus* Harms, ASH) is a wild deciduous shrub that grows in the northern cold regions of Hokkaido in Japan and China. ASH has been widely used as a Chinese herbal medicine and has recently been marketed as a dietary supplement in Japan and Western countries. ASH contains several active ingredients, including phytochemicals, which exhibit therapeutic effects on diabetes,^11^ allergies,^12^ gastric ulcers,^13^ neurodegenerative disease,^14^ and cancer.^15^ The root of ASH is also known to have a suppressive effect on inflammation and oxidative stress,^16^ suggesting the advantage of the aforementioned effects on CRF. Here, we conducted a feasibility study to examine how many patients would be able to complete a 28-day course of CRF treatment with ASH.

## Patients and Methods

This study included patients who had advanced stage cancer and were either outpatients or hospitalized. The detailed inclusion and exclusion criteria for the patient are shown in Supplementary Table S1. Several parameters of the patients enrolled in this study were examined as shown in Supplementary Table S2 (prestudy). Subsequently, they were instructed to consume a drink consisting of the ASH root extract (Sun Eleuthero Extract; Sun Chlorella Co., Ltd.) at a volume of 30 mL twice daily for continuous 28 days. After completion of ASH extract drinking, poststudy was conducted similar to the prestudy. The primary endpoint was the proportion of patients who were able to complete the brief fatigue inventory (BFI) survey on poststudy (study completion rate), and secondary endpoints were the degree of improvement in CRF-related parameters (BFI, diacron-reactive oxygen metabolites (dROMs), biological antioxidant potential (BAP), and IL-6), and adverse events during the study. Data on adverse events were obtained from patients’ medical records. The target enrollment was 15 patients, and the study was conducted between March 18, 2021, and March 8, 2022. Informed consent was obtained by all enrolled patients.

Statistical evaluations for comparison between prestudy and poststudy included a nonparametric paired (Wilcoxson signed-rank sum) and nonpaired *t*-test using SPSS (version 25, IBM Corp., Armonk, NY).

## Results

Patient clinical information is summarized in Table 1. The median age of the patients was 76 (52–87) years, nine were female and six were male. The most common primary cancer site was colon in six cases, followed by stomach in three cases, lung and pancreas in two cases, and bladder and ovarian in one case each. Two patients (EUR-04, 08) had stage III disease in TNM classification, and the remaining 13 patients had more advanced stage IV disease. At enrollment (prestudy), BFI in all 15 patients had a median value of 6.4 (3.8–9.3), IL-6 had a median value of 21.6 (2.6–103.0) pg/ml, dROMS had a median value of 369 (143–653) U.CARR, and BAP had a median value of 1882 (1206–2366) μmol/l. Nine patients drank the ASH extract for 28 days, whereas the remaining six patients could not complete the study duration. Among these nine patients, seven patients (EUR-01, 05, 06, 08, 13, 14, and 15) completed the BFI survey (study completion rate; 46.7%) at poststudy. The remaining two patients were unable to complete the BFI survey at poststudy owing to either the deterioration of sepsis (EUR-02) or protocol deviation (EUR-04) (Table 2). Of the remaining six patients who were unable to complete the study duration, five had worsening complications [pneumonia, bowel (intestinal) obstruction, interstitial pneumonia, liver failure, and trousseau syndrome, respectively], and the family member of one patient requested discontinuation of the study. Patients with lower BFI as well as lower eastern oncology cooperative group-performance status (ECOG-PS) scores at prestudy completed the study (BFI: 5.7 ± 1.6 for completed seven cases, 7.2 ± 1.7 for discontinued nine cases, *p* = 0.04; ECOG-PS: 0.6 ± 0.5 for completed seven cases, 1.8 ± 1.0 for discontinued nine cases, *p* = 0.02).

**Table 1.** Clinical Information of Enrolled Patients in This Study

					Prestudy						
Patient	Age	Sex	Cancer type	Stage	BFI	ECOG-PS	IL-6 (pg/ml)	dROMS (U.CARR)	BAP (μmol/l)	dROMS/BAP	ASH drinking period (days)
EUR-01	76	M	Cecum	IVC	8.1	1	22.4	372	1746	4.69	28
EUR-02	85	F	Bladder	IVB	7.9	2	18.7	369	2150	5.82	28
EUR-03	77	M	Lung	IVA	6.4	3	21.6	452	1947	4.30	4
EUR-04	87	F	Colon	IIIB	3.8	0	2.5	311	1662	5.34	28
EUR-05	82	F	Lung	IVB	7.4	1	8.3	309	1764	5.70	28
EUR-06	52	F	Stomach	IVB	5.3	0	5.4	269	1561	5.80	28
EUR-07	78	F	Ovary	IV	7.2	2	77.8	229	2147	9.37	19
EUR-08	83	F	Pancreas	III	3.8	1	5.5	491	2020	4.11	28
EUR-09	68	F	Rectum	IVC	7.9	1	58.7	571	2050	3.59	15
EUR-10	79	M	Stomach	IVB	8.8	3	81.1	150	1657	11.04	24
EUR-11	59	M	Rectum	IVA	6.4	2	53.8	471	1882	3.99	15
EUR-12	61	F	Stomach	IVB	9.3	1	26.8	143	1206	8.43	14
EUR-13	75	F	Pancreas	IV	4.7	1	2.6	254	1669	6.57	28
EUR-14	64	M	Colon	IVC	5.8	0	17.1	377	2290	6.07	28
EUR-15	55	M	Rectum	IVC	4.6	0	103	653	2366	3.62	28

ASH, Acanthopanax senticosus Harms; BAP, biological antioxidant potential; BFI, brief fatigue inventory; dROMS, diacron-reactive oxygen metabolites; ECOG-PS, eastern cooperative oncology group-performance status.

**Table 2.** Reasons for Discontinuance and Adverse Events During This Study

Patient	Reason for discontinuance	Adverse events			
EUR-01	—	Diarrhea			
EUR-02	Sepsis	Fever	Arthritis		
EUR-03	Pneumonia	Dyspnea			
EUR-04	Protocol deviation	Malaise			
EUR-05	—	Nausea	Malaise		
EUR-06	—	Edema limbs (lower)	Hypersomnia	Arthralgia	Numbness (foot)
EUR-07	Family’s cancel request	Bloating	Stomach pain		
EUR-08	—				
EUR-09	Bowel obstruction	Vomiting			
EUR-10	Interstitial pneumoniae	Dyspnea			
EUR-11	Liver failure	Depressed level of consciousness			
EUR-12	Trousseau syndrome	Oculomotor nerve disorder	Pain (upper limb)	Dysesthesia (upper limb)	
EUR-13	—				
EUR-14	—				
EUR-15	—				

Supplementary Table S3 presents the results of blood count and biochemical tests in seven patients who completed the study. No significant changes were observed between pre- and poststudies.

**FIG. 1.**
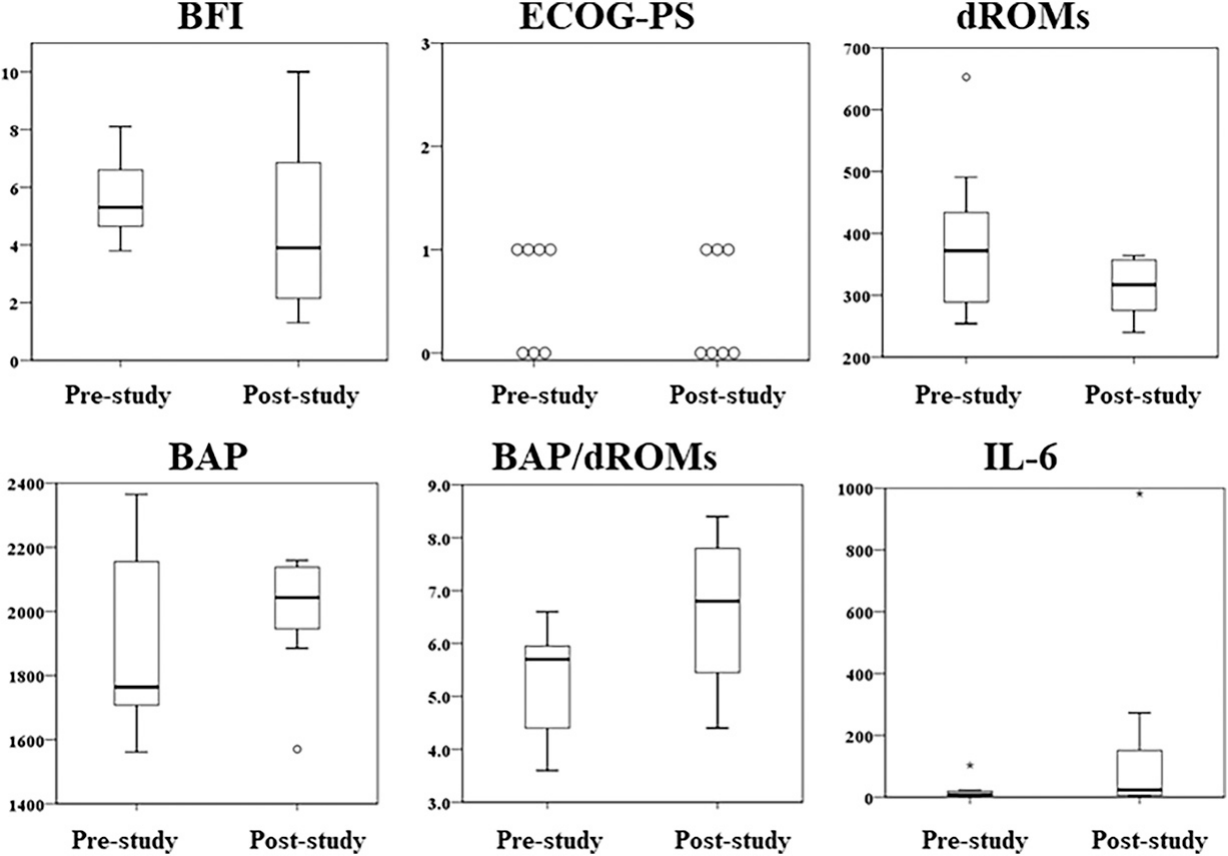
Box plot (BFI, dROMs, BAP, BAP/dROMs, and IL-6) and Dot plot (ECOG-PS). BFI, brief fatigue inventory; dROMS, diacron-reactive oxygen metabolites; BAP, biological antioxidant potential.

We further examined the changes in CRF-associated parameters in seven patients who completed the study (Fig. 1 and Supplementary Table S4). Four patients with BFI score at prestudy <5.5, corresponding to median value, showed a decrease in BFI score poststudy, whereas only one patient (EUR-01) out of three who had BFI >5.5 showed a decrease poststudy. Overall BFI score was also decreased (median value: prestudy: 5.3; poststudy: 3.9) but not significant (*p* = 0.398). The value of dROMs decreased and also both the value of BAP and ratio of BAP to dROMS (BAP/dROMs)—the potential antioxidant capacity increased, but all these changes were not significant (dROMs, *p* = 0.298; BAP, *p* = 0.499; BAP/dROMs, *p* = 0.063). The increased value of IL-6 was not significant (*p* = 0.063).

Eleven patients reported several adverse events during the study (Table 2), whereas the remaining four patients (EUR-08, 13, 14, 15) who completed the study had no adverse events.

## Discussion

In this feasibility study, 9 out of 15 patients with advanced cancer successfully consumed the ASH extract for 28 days. The percentage of patients who completed the BFI at poststudy was 46.7%, and most patients discontinued the study owing to the exacerbation of complications.

Regarding safety, several adverse events were observed (Table 2). Because the patients for whom ASH consumption was likely to worsen existing diseases and complications were excluded at the time of enrollment, the physician-in-charge considered a clear causal relationship between adverse events and the ASH extract to be negative. However, we could not rule out the possibility of association with adverse events owing to ASH; therefore, attention is to be paid to this point in further studies.

Four patients whose BFI score was <5.5 at prestudy experienced an improvement in CRF, whereas the remaining patients whose BFI score was >5.5 had little effect on CRF. Also, the lower the BFI and ECOG-PS scores, the more patients completed the study, suggesting that ASH extract consumption exerts a certain effect on mild CRF in patients who have better functional status and that these patients are less likely to drop out of the study owing to cancer-related complications.

Oxidative stress markers and inflammatory cytokines are elevated in patients with advanced cancer and are associated with CRF.^6,7^ A previous study reported that dROMs, BAP, and BAP/dROMs were associated with both oxidative stress^17^ and fatigue in healthy volunteers.^18^ ASH was reported to have both antioxidant and antiinflammatory effects.^16^ In our study, antioxidant parameters, such as the value of BAP and the ratio of BAP/dROMs, showed an increase to some extent, suggesting that in addition to the antioxidant capacity, ASH also had an alleviating effect on CRF in patients with advanced cancer. However, no significant decrease in serum IL-6 was observed, indicating that ASH extract had few effects on IL-6 production in cancer. A previous study reported that a reduction by 2 points on the BFI score was considered an important clinical difference to prove the clinical efficacy of CRF improvement.^19^ Our study showed a reduction of 1.4 points in the BFI score even in only seven cases (Supplementary Table S4), and further clinical studies are warranted to assess the effect of ASH on not only CRF but also the cytokine milieu by enrolling more subjects.

The limitation of this study is that it is a feasibility study; therefore, more cases are needed to verify the efficacy of ASH extract. Moreover, the relatively short duration of ASH extract consumption (28 days) is considered to limit not only the efficacy but also the adverse events.

To summarize, 46.7% represents the number of patients who completed the follow-up BFI survey after a 28-day course of ASH, whereas 9 out of 15 patients completed the 28-day course of ASH. Seven patients who completed the study not only had an improvement in the mild CRF but also an increase in the antioxidant stress capacity. As ASH extract is readily available, this might help the patient with relieving mild CRF by consuming it as a supplementary food. In future, long-term and large-scale study needs to be performed to validate the usefulness of ASH extract in a patient population with mild CRF and good general condition.

## Abbreviations Used

### Abbreviations Used

ASH: Acanthopanax senticosus Harms
BAP: Biological antioxidant potential
BFI: Brief fatigue inventory
CRF: Cancer-related fatigue
dROMs: Diacron-reactive oxygen metabolites
