# Supplementary material for: Feasibility and Safety of Food Containing Acanthopanax senticosus for Treating Patients with Cancer-Related Fatigue
Source: Palliat Med Rep. 2024 Aug 23;5(1):381–6. doi: 10.1089/pmr.2024.0041 (PMC11392680; doi:10.1089/pmr.2024.0041)
Supplement: Supplementary Table S1 [file pmr.2024.0041_kawano_supplementary_table_1.pdf]

Supplementary Table 1 Patient inclusion/exclusion criteria

Inclusion criteria

prognosis assumed to be more than 1 month

able to take an oral intake sufficiently

score of Brief Fatigue Inventory (BFI) three or more

age 20 years or older at the time of informed consent

Exclusion criteria

consume any ASH-containing food within the past 3 months before registration

allergic to ASH-containing food

estrogen receptor positive cancer, such as breast, uterine, and ovary

undergone receiving chemotherapy with doxyfluoridine

hypertension greater than 180/90 mmHg

digoxin medication

pregnant, possibly pregnant, or lactating women

endometriosis or myoma uteri

mental disorders.
