# Supplementary material for: Feasibility and Safety of Food Containing Acanthopanax senticosus for Treating Patients with Cancer-Related Fatigue
Source: Palliat Med Rep. 2024 Aug 23;5(1):381–6. doi: 10.1089/pmr.2024.0041 (PMC11392680; doi:10.1089/pmr.2024.0041)
Supplement: Supplementary Table S2 [file pmr.2024.0041_kawano_supplementary_table_2.pdf]

Supplementary Table 2 Parameters examined in this study

General status

Eastern Cooperative Oncology Group Performance status: ECOG-PS

Cancer related fatigue

Brief Fatigue Inventory: BFI

Physical examination

Height

Weight

Blood pressure

Pulse

Body temperature

Peripheral blood test

Blood cell

White blood cell: WBC

Red blood cell: RBC

Platelet: Plt

Biochemical tests

total protein: TP

Albumin: Alb

Aspartate aminotransferase: AST

Alanine aminotransferase: ALT

Lactate dehydrogenase: LDH

Alkaline phosphatase: ALP

γ-glutamyl transpeptidase: γ-GTP

Total bilirubin

Creatinine: Cr

Blood urea nitrogen: BUN

Uric acid: UA

Creatinine phosphokinase: CPK

Total cholesterol: T.Chol

Triglyceride: TG

Sodium: Na

Potassium: K

Chloride: Cl

Fasting blood sugar: BS

	C-reactive protein: CRP
Cytokine and stress marker	
	IL-6
	Diacron-Reactive Oxygen Metabolites: dROMs
	Biological Anti-Oxidant Potential: BAP
