# Supplementary material for: Feasibility and Safety of Food Containing Acanthopanax senticosus for Treating Patients with Cancer-Related Fatigue
Source: Palliat Med Rep. 2024 Aug 23;5(1):381–6. doi: 10.1089/pmr.2024.0041 (PMC11392680; doi:10.1089/pmr.2024.0041)
Supplement: Supplementary Table S3 [file pmr.2024.0041_kawano_supplementary_table_3.pdf]

Supplementary Table 3 Changes in blood tests between pre and post study

	Pre-study		Post-study		t test
	Average	SD	Average	SD	pre vs post
TP (g/dl)	6.1	0.8	6.2	0.7	0.886
Alb (g/dl)	3.2	0.3	3.3	0.3	0.815
AST (U/l)	28.4	8	26.9	7.2	0.705
ALT (U/l)	27.7	16.8	16.1	7.6	0.123
LDH (U/l)	256.3	83.3	390.6	378.7	0.378
ALP (U/l)	279.3	344.2	217.3	298.9	0.725
γGTP (U/l)	278.3	383	216.6	353.2	0.759
T. bil (mg/dl)	1.4	2	0.6	0.3	0.341
Cr (mg/dl)	0.8	0.3	0.8	0.3	0.977
BUN (mg/dl)	16.9	4.3	19.3	5.5	0.376
UA (mg/dl)	5.2	1.1	5.9	2.3	0.446
CPK (U/l)	62.4	38.9	78	51.6	0.536
T. Chol (mg/dl)	182.9	47	187.6	89.1	0.904
TG (mg/dl)	113.9	60.5	112.9	38.9	0.971
Na (mEq/l)	137.9	3.5	138.7	1.8	0.578
K (mEq/l)	4.4	0.5	4.1	0.5	0.372
Cl (mEq/l)	102	5.7	103.3	4.1	0.637
BS (mg/dl)	101.1	16.5	101	9.2	0.984
WBC (μ/l)	6667.1	2700.4	7747.1	6027.3	0.673

**RBC ($x10^4$ /µl)**	**359.6**	**88.8**	**348.3**	**86.2**	**-11.3**	**41.3**	**0.813**
**Hb (g/dL)**	**11.1**	**2.7**	**10.9**	**2.5**	**-0.2**	**1.1**	**0.873**
**Hct (%)**	**34.2**	**7.7**	**33.8**	**8**	**-0.4**	**3.4**	**0.918**
**Plt ($x10^4$ /µl)**	**28.6**	**6.8**	**21.9**	**8.7**	**-6.7**	**7.2**	**0.135**
**CRP (mg/dL)**	**1.7**	**2.5**	**5.4**	**11.2**	**3.7**	**8.9**	**0.411**

SD, standard deviation

TP, total protein; Alb, Albumin; AST, aspartate aminotransferase; ALT, alanine aminotransferase

LDH, lactate dehydrogenase; ALP, alkaline phosphatase; γ-GTP, γ-glutamyl transpeptidase; T.bil, total bilirubin; Cr, creatinine

BUN, blood urea nitrogen; UA, uric acid; CPK, creatinine phosphokinase; T. Chol, total cholesterol; TG, triglyceride

Na, Sodium; K, Potassium; Cl, Chloride; BS, fasting blood sugar; WBC, white blood cell; RBC, red blood cell; Plt, platelet; CRP, C-reactive protein.
