# Supplementary material for: Feasibility and Safety of Food Containing Acanthopanax senticosus for Treating Patients with Cancer-Related Fatigue
Source: Palliat Med Rep. 2024 Aug 23;5(1):381–6. doi: 10.1089/pmr.2024.0041 (PMC11392680; doi:10.1089/pmr.2024.0041)
Supplement: Supplementary Table S4 [file pmr.2024.0041_kawano_supplementary_table_4.pdf]

Supplementary Table 4 Changes in CRF-associated parameters between pre and post study

	BFI		ECOG-PS		dROMs		BAP		BAP/dROMs		IL-6	
Patient	Pre-Study	Post-Study	Pre-Study	Post-Study	Pre-Study	Post-Study	Pre-Study	Post-Study	Pre-Study	Post-Study	Pre-Study	Post-Study
EUR-01	8.1	4.4	1	0	372	258	1746	2127	4.7	8.2	22.4	29.1
EUR-05	7.4	10.0	1	1	309	317	1764	2149	5.7	6.8	8.3	8.1
EUR-06	5.3	1.3	0	0	269	355	1561	1570	5.8	4.4	5.4	23.5
EUR-08	3.8	2.1	1	1	491	364	2020	2043	4.1	5.6	5.5	5.1
EUR-13	4.7	3.9	1	1	254	240	1669	2006	6.6	8.4	2.6	3.3
EUR-14	5.8	9.3	0	0	377	293	2290	2159	6.1	7.4	17.1	273
EUR-15	4.6	2.2	0	0	653	359	2366	1885	3.6	5.3	103	982
Median	5.3	3.9	1.0	0.0	372.0	317.0	1764.0	2043.0	5.7	6.8	8.3	23.5
Average	5.7	4.8	0.6	0.4	389.3	312.3	1916.6	1991.3	5.2	6.6	23.5	189.2
SD	1.6	3.5	0.5	0.5	141.2	50.4	314.2	209.4	1.1	1.5	35.8	362.8

SD, standard deviation

BFI, Brief Fatigue Inventory; ECOG-PS, Eastern Cooerative Oncology Group performance status

dROMS, Diacron-Reactive Oxygen Metabolites; BAP, Biological Anti-Oxidant Potential
